# Supplementary material for: A selection and optimization strategy for single-domain antibodies targeting the PHF6 linear peptide within the tau intrinsically disordered protein
Source: J Biol Chem. 2024 Mar 12;300(4):107163. doi: 10.1016/j.jbc.2024.107163 (PMC11007443; doi:10.1016/j.jbc.2024.107163)
Supplement: Supporting Figures 1–11 [file mmc1.pdf]

Supporting Information

R47I	S23C A64V	P101S G115E
R47I	S23C G115E	F13Y R47K E48A
R47I	A25S R47K	S23I R47K E56D
R47K	A42V G115E	S23C G58V G115E
R47K	K45N G115E	S23C G58V G115E
T96S	R47K S59L	S23C G58V G115E
T96S	R47K Y97C	S23N K79E G115E
P101S	R47I P101L	T32I E56G G115E
G115E	A64V G115E	R47K Y113N Q116H
G115E	D65V G115E	R90G P101S G114E
G115E	D65N G115E	A8G Q15R M36L R47I
G115E	D76V G115E	S27A N77S V82A T96S
Q15H N80T	N77K G115E	G58E N80T Y83S G115E
G17E G115E	S78P G115E	
S23C R47I	K79E N80T	

Figure S1 List of 43 mutants obtained from the initial yeast two-hybrid screen.

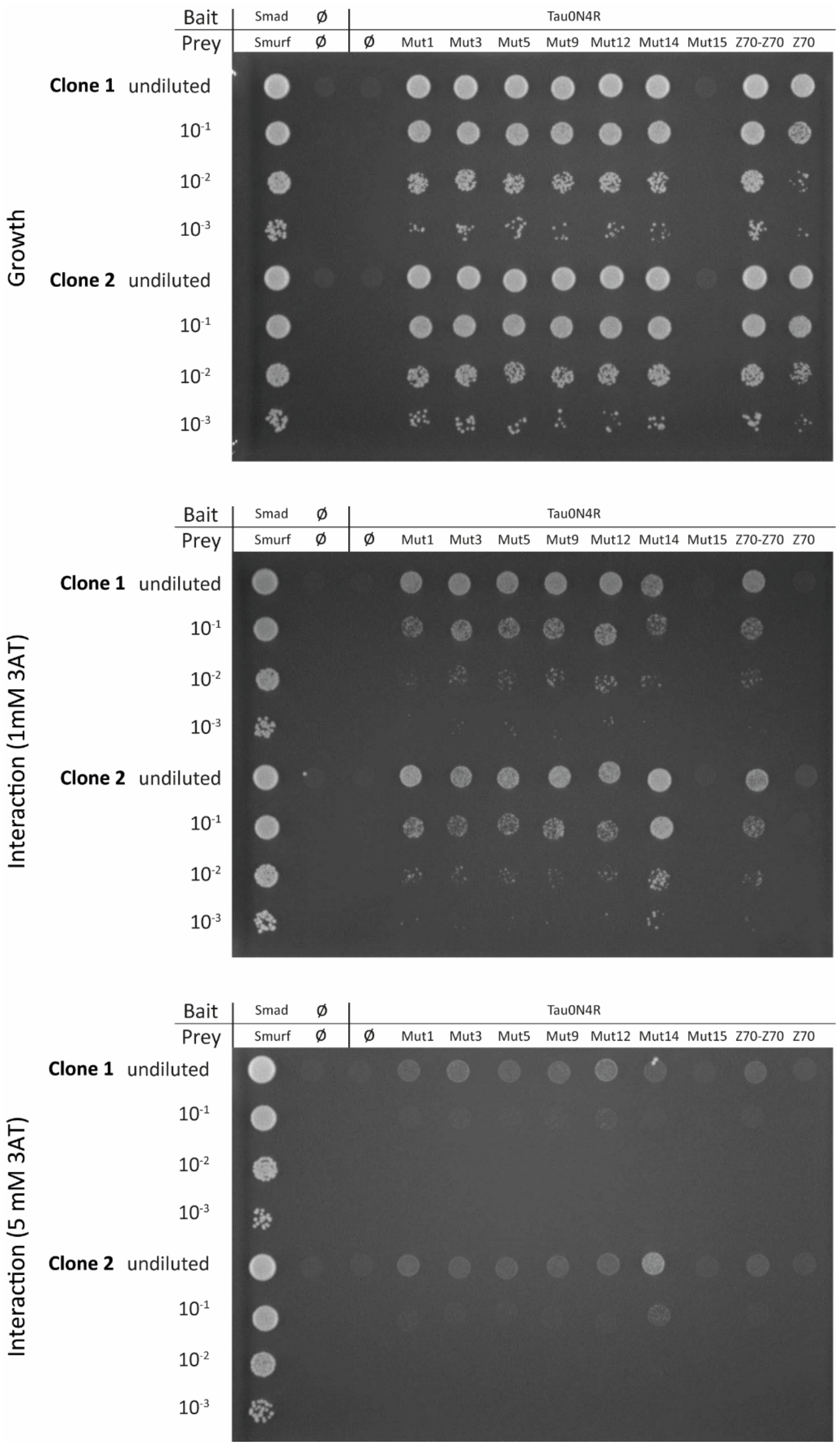

Fig S2 One-to-one mating by yeast two-hybrid on selective medium without tryptophane and leucine (growth) and without tryptophane, leucine and histidine and in the presence of 1 mM or 5 mM 3AT (interaction). Yeast growth is monitored by serial dilutions and for 2 distinct clones. Ø represents an empty vector. Z70-Z70 represents two linked VHHs Z70 VHHs.

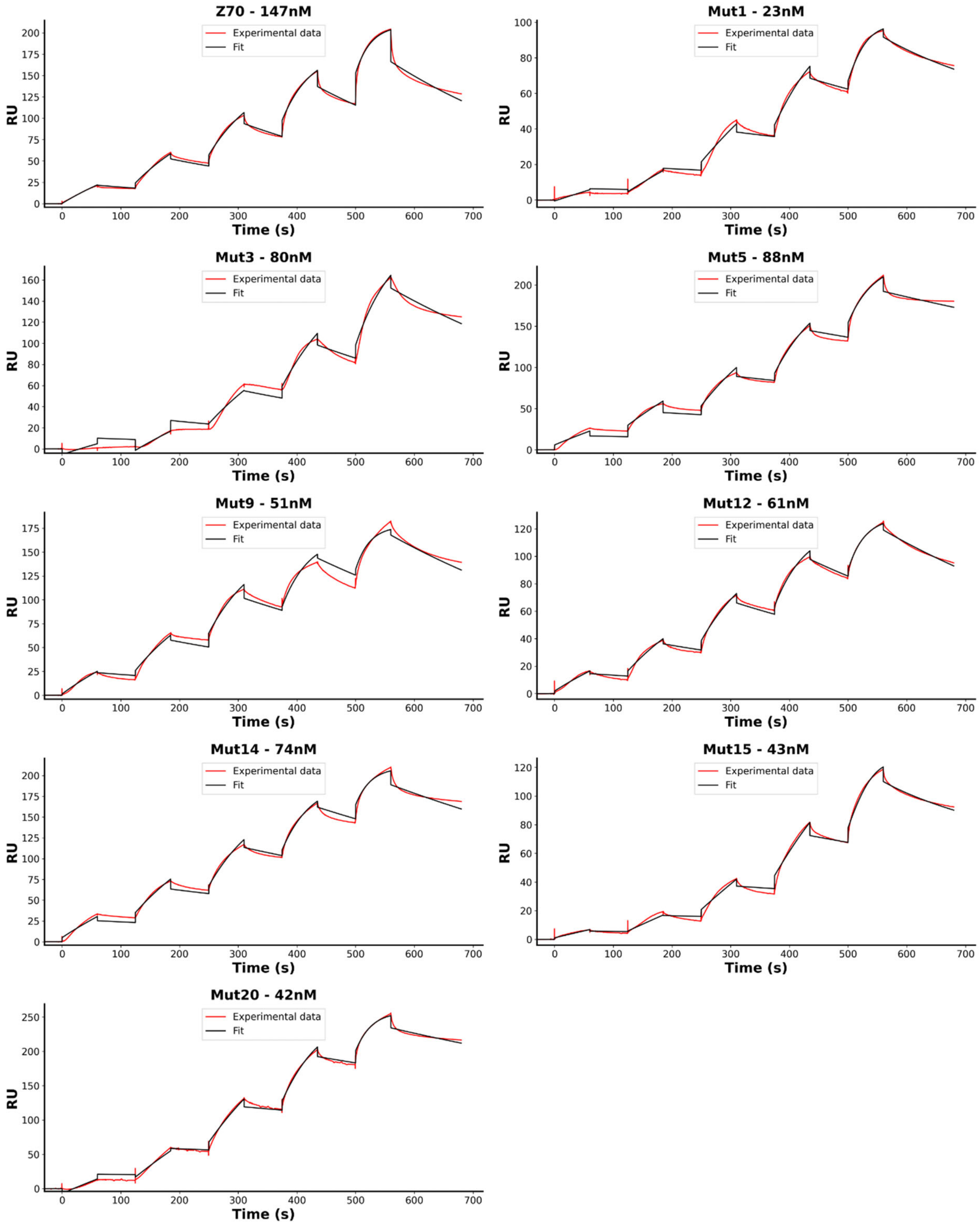

Figure S3 Sensorgrams of serial injections of VHHs (0.125, 0.25, 0.5, 1 and 2 µM) on a Tau immobilized SA chip in red and fitting with a 1:1 model in black.

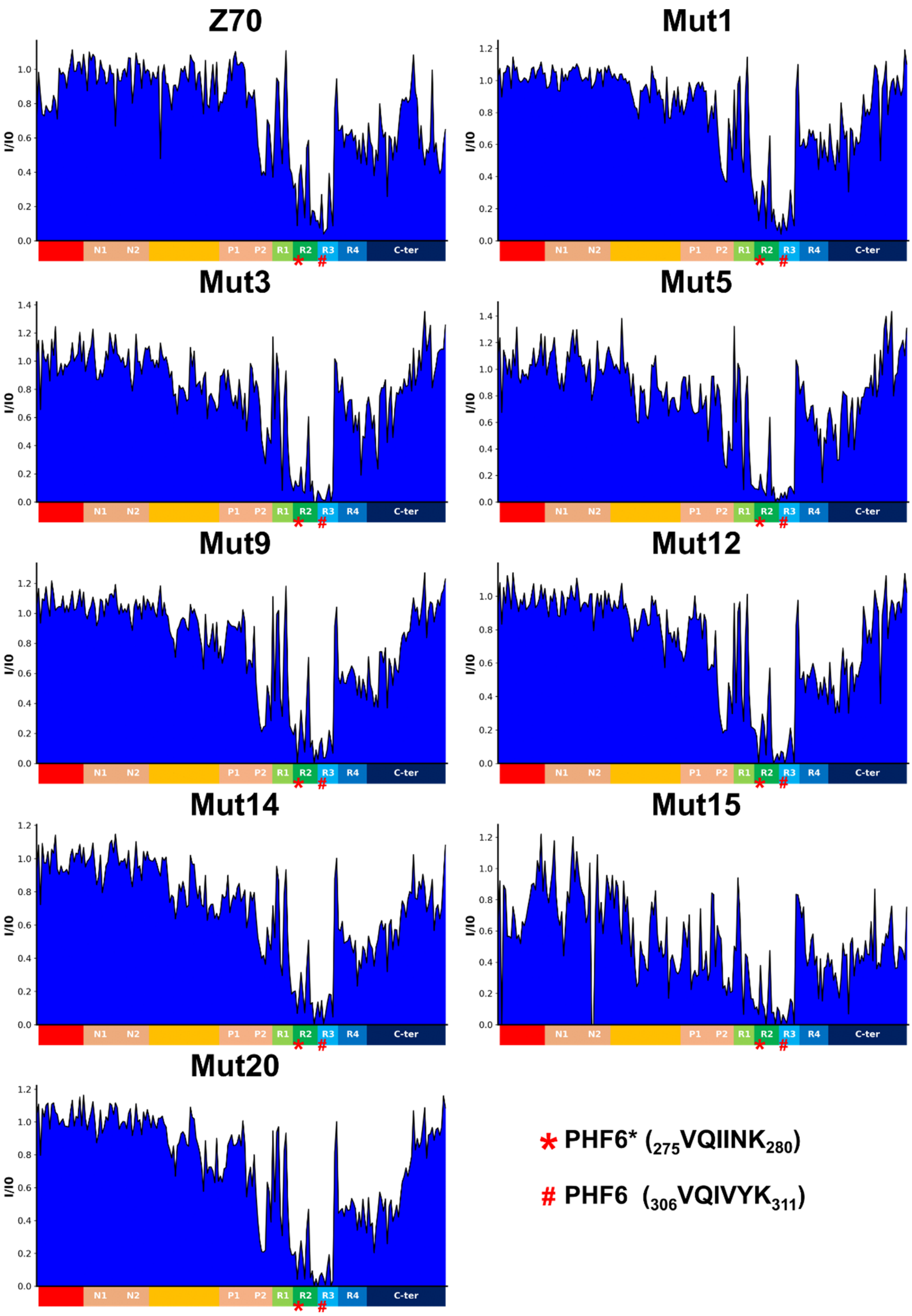

Figure S4 Normalized intensity ratios I/I0 of corresponding resonances in the two-dimensional spectra of Tau with equimolar quantity of the different VHHs (I) or free in solution (I0) for residues along the Tau sequence. Data corresponding to Z70, Mut1, Mut3 and Mut 20 are here duplicated from Fig.3B to facilitate comparison with the other mutants.

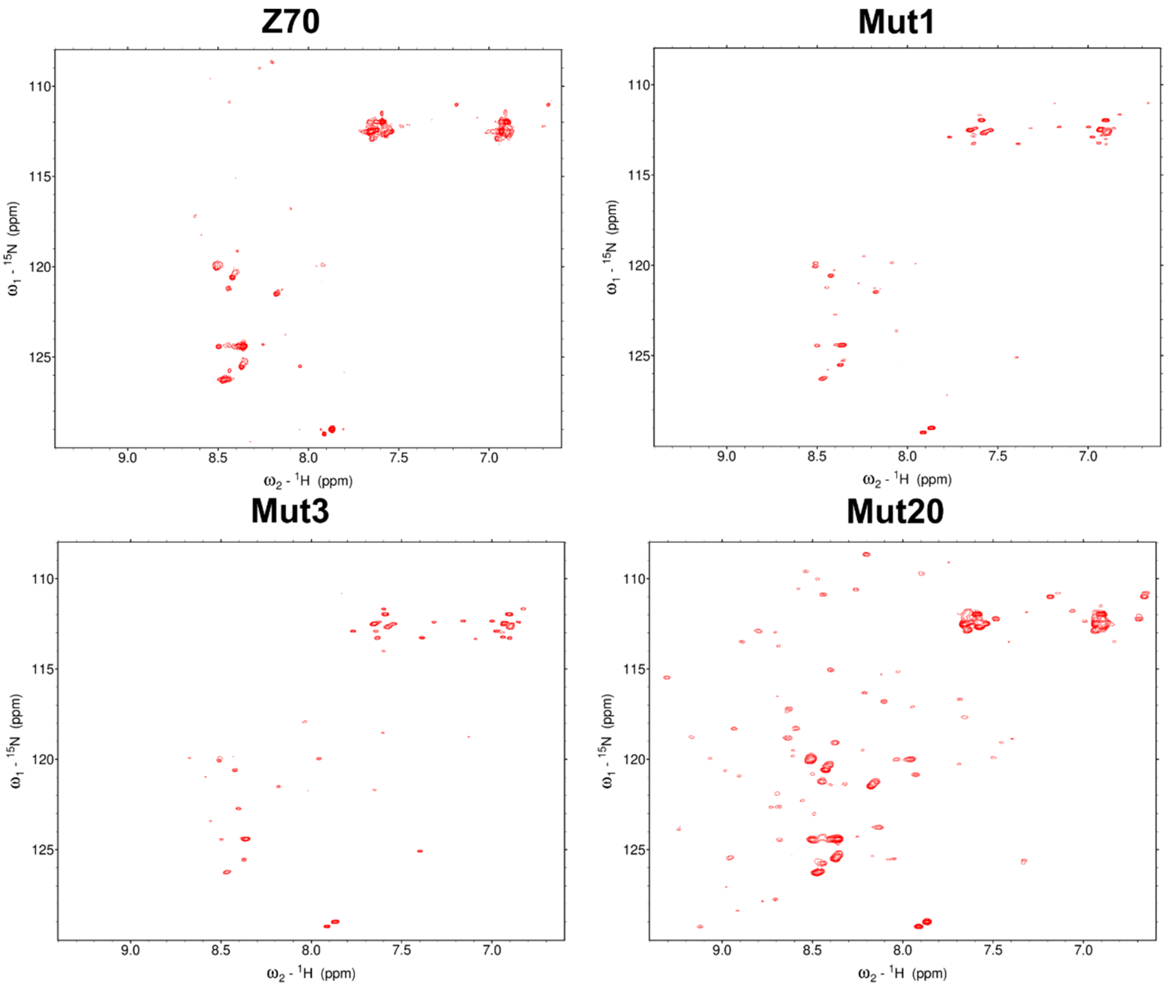

Fig S5 ^{1}H ^{15}N HSQC spectra of ^{15}N labeled Z70, mutant 1, mutant 3 and mutant 20. The quality of these spectra was poor as the number of ^{1}H ^{15}N resonances that were detected were well below what could be expected based on the amino-acid sequence. However, mutant 20 showed comparatively more resonances compared to the other VHHs, which can be attributed to a modification of its conformational dynamics.

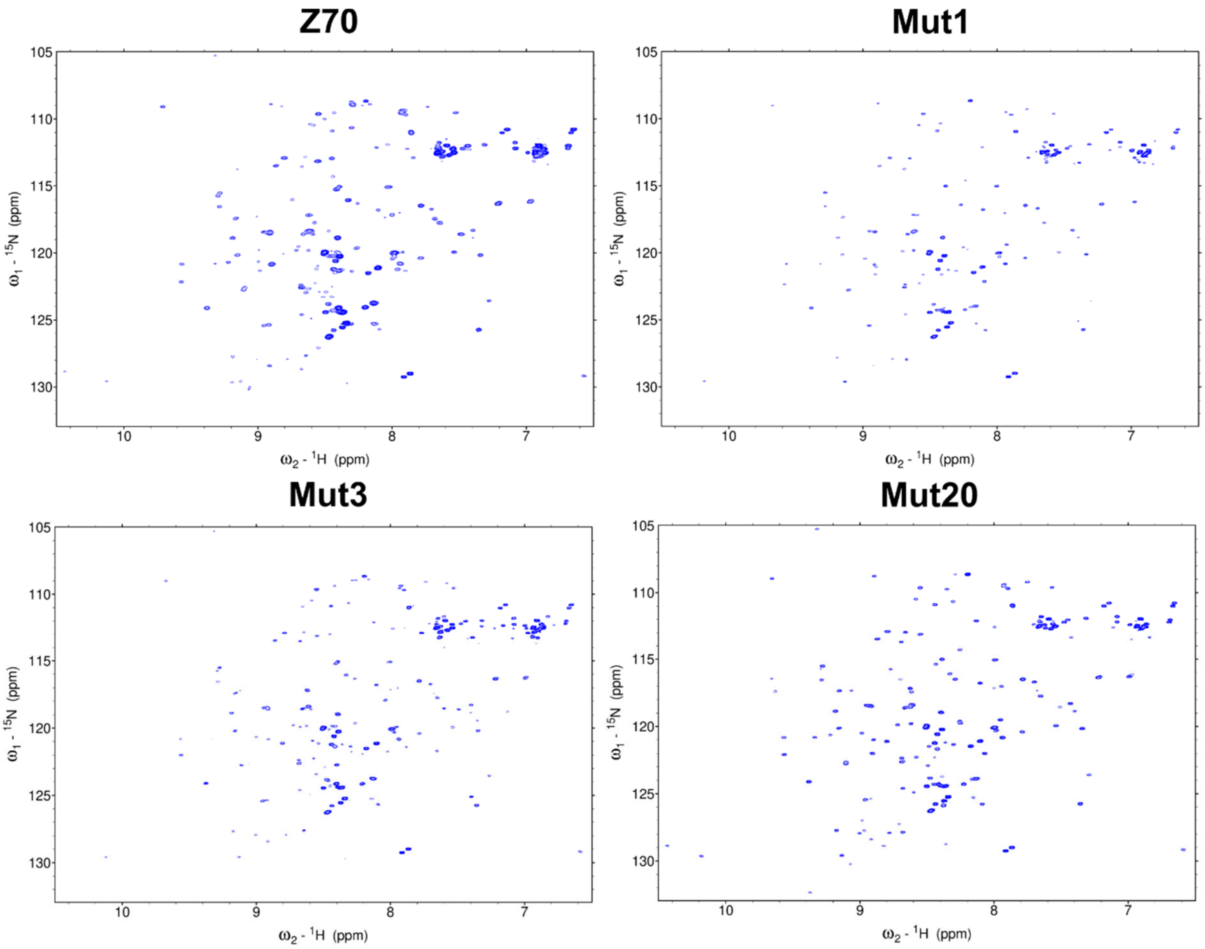

Fig S6 ^{1}H ^{15}N HSQC spectra acquired in identical conditions of ^{15}N labeled Z70, mutant 1, mutant 3 and mutant 20 at the same concentration (100 µM) in the presence of a two-fold excess of the PHF6 peptide. All VHHs showed comparatively more resonances compared to Fig. S5, which can be attributed to a modification of the conformational dynamics when bound to the PHF6 peptide. Mutant 20 bound to the PHF6 peptide showed the best spectrum quality.

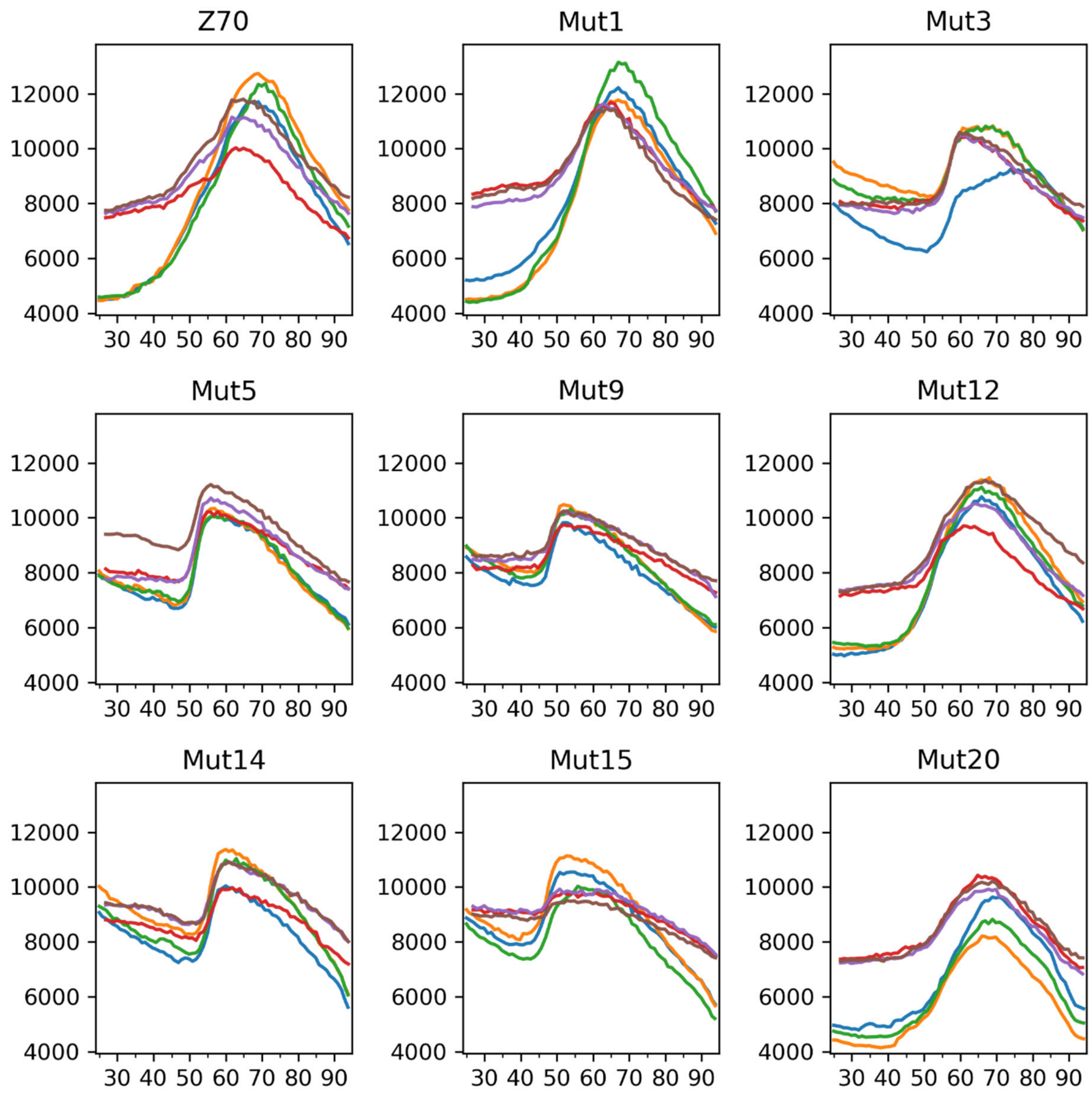

Fig S7 Superposition of the raw fluorescence values from TSA experiments for the 9 tested VHHs from 2 independent experiments with 3 replicates (experiment 1: blue, green, orange and experiment 2: red, brown, purple curves).

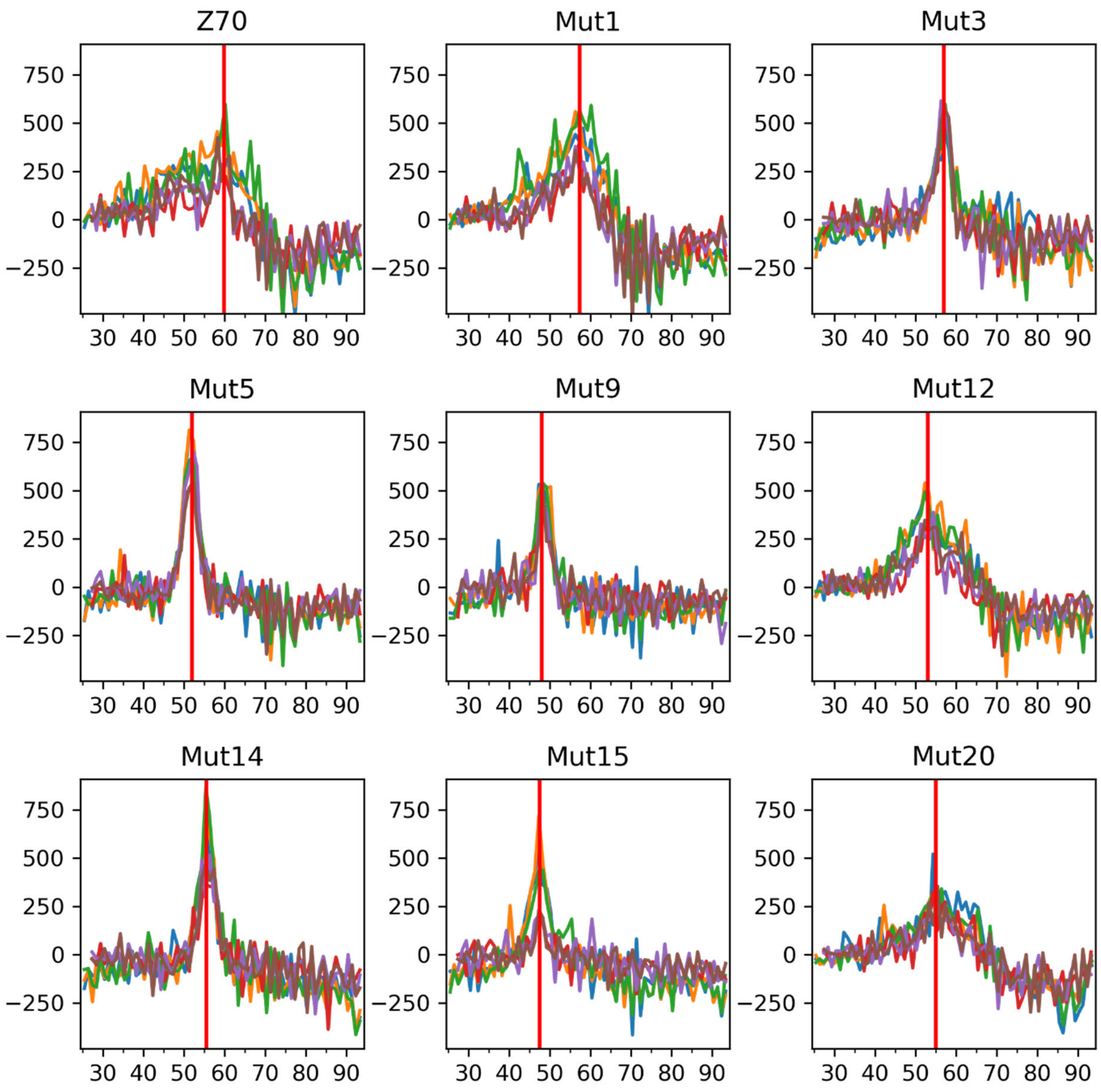

Fig S8 Superposition of the derived fluorescence values from TSA experiments for the 9 tested VHHs from 2 independent experiments with 3 replicates (experiment1 : blue, green, orange and experiment2 : red, brown, purple curves) used for melting temperatures determination, shown as a vertical red line.

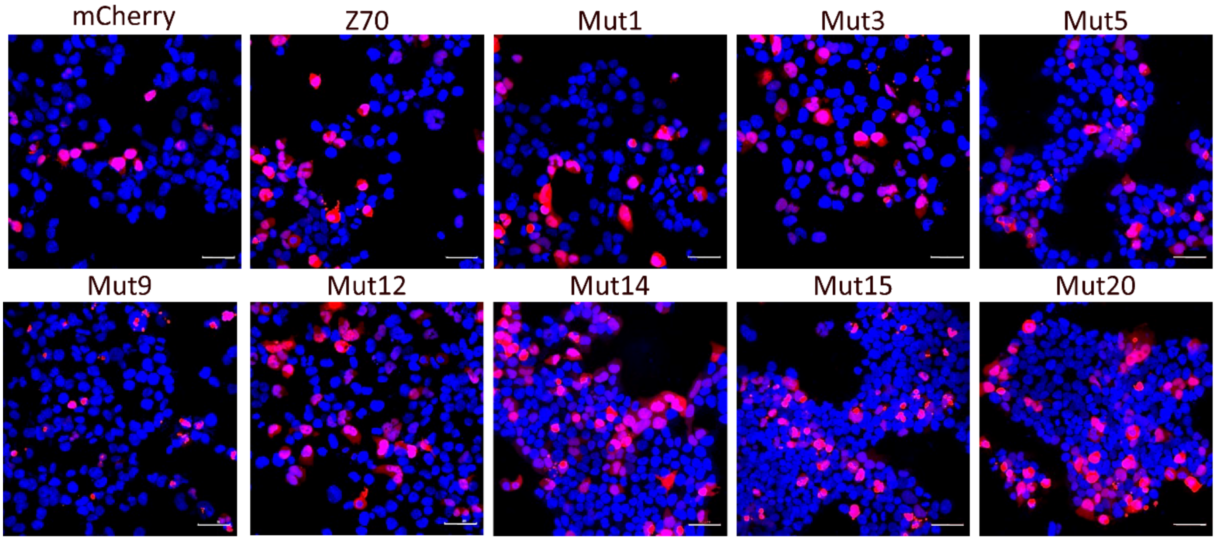

Fig S9 Self-aggregation of the mCherry constructs. The presence of aggregates is noticed by the appearance of "puncta" inside the cells in contrast with an uniform fluorescence across the cell in the absence of aggregates. The white scale bar corresponds to 50 μm.

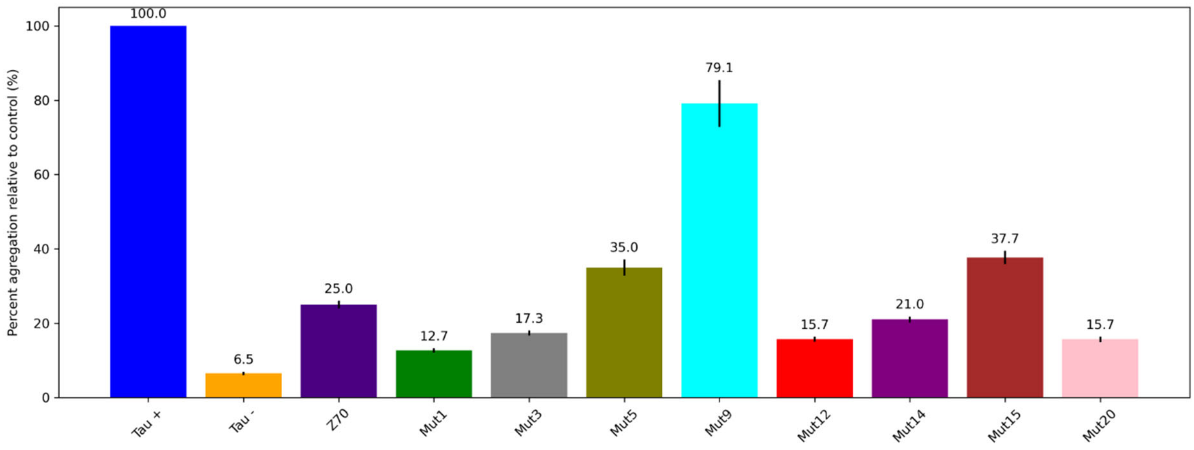
Percent agregation relative to control (%)
100.0
6.5
25.0
12.7
17.3
35.0
79.1
15.7
21.0
37.7
15.7
Tau +
Tau -
Z70
Mut1
Mut3
Mut5
Mut9
Mut12
Mut14
Mut15
Mut20

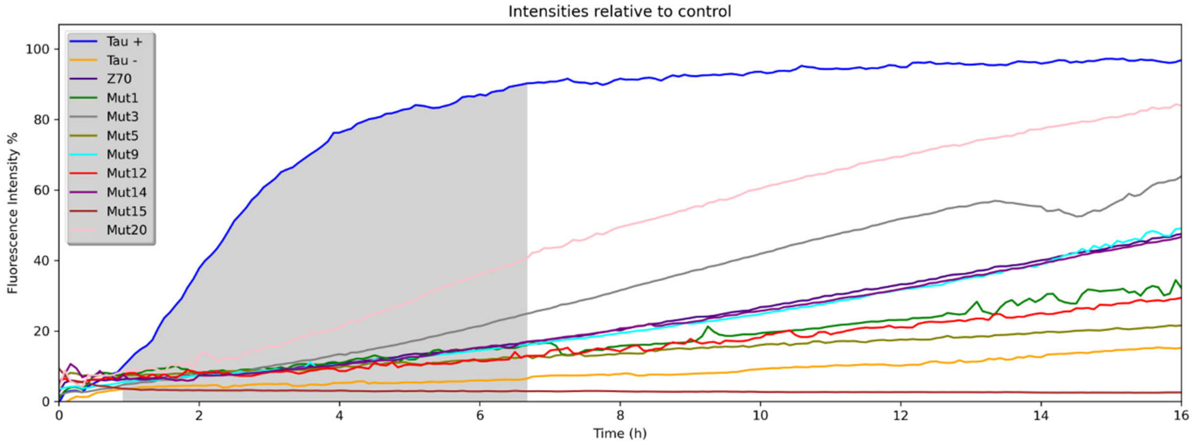
Intensities relative to control
Fluorescence Intensity %
Time (h)
Tau +
Tau -
Z70
Mut1
Mut3
Mut5
Mut9
Mut12
Mut14
Mut15
Mut20

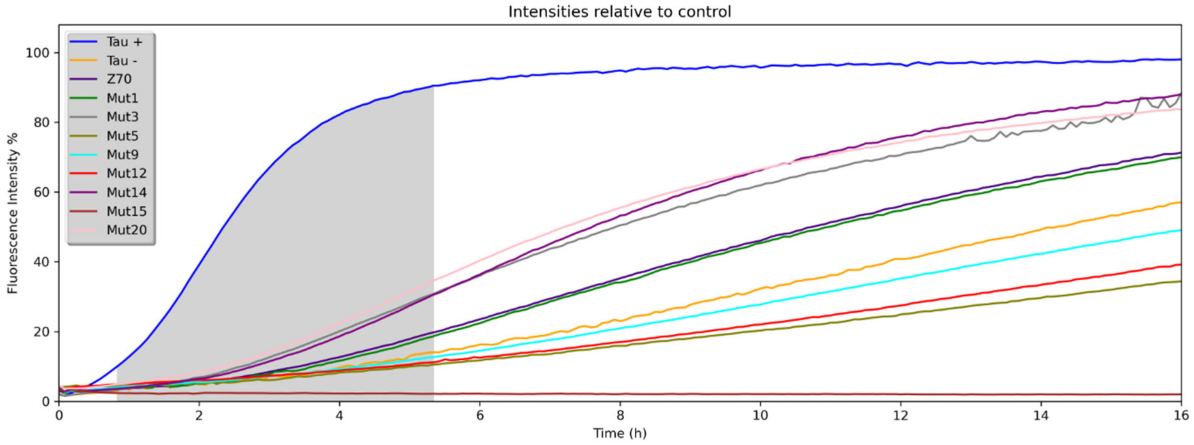
Intensities relative to control
Fluorescence Intensity %
Time (h)
Tau +
Tau -
Z70
Mut1
Mut3
Mut5
Mut9
Mut12
Mut14
Mut15
Mut20

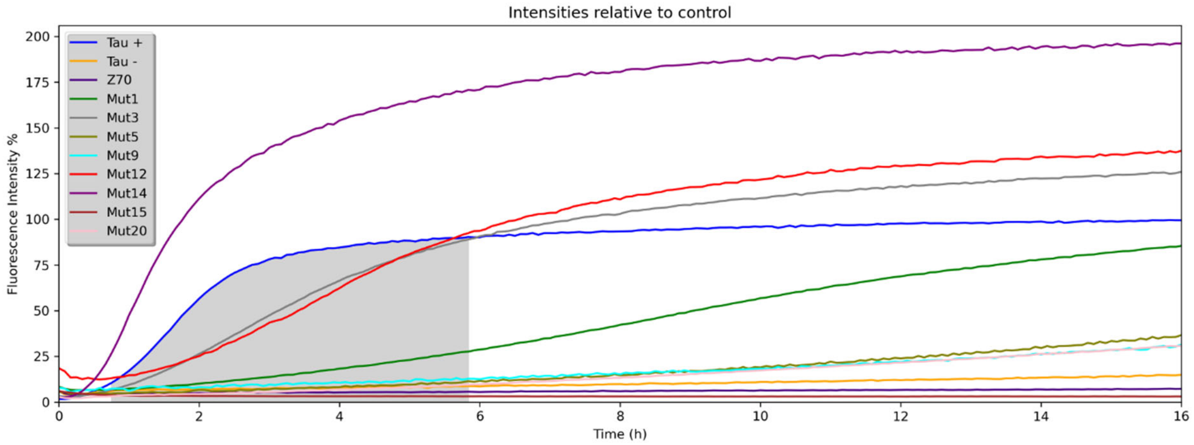
Intensities relative to control
Fluorescence Intensity %
Time (h)
Tau +
Tau -
Z70
Mut1
Mut3
Mut5
Mut9
Mut12
Mut14
Mut15
Mut20

Figure S10 Inhibition efficiency of different Z70-derived VHHs over Tau aggregation tested at a 0.5:1 VHH:Tau molar ratio. Positive and negative controls correspond to 10 µM Tau in the presence of 2.5 mM heparin (Tau +) and the absence of heparin (Tau -), respectively. The other conditions correspond to 10 µM Tau in the presence of 2.5 mM heparin and the different VHHs at 5 µM. **A.** The percentage of aggregation corresponds to the fluorescence intensity ratio between Tau in the presence of a VHH and Tau alone (Tau +, 100% aggregation) during the aggregation process. B. Intensity curves from *in vitro* aggregation assays from 3 independent experiments. Data are represented as mean ± sem, the mean value is written above the bars. The gray area corresponds to the 10-90% dynamic range of fluorescence intensity measured for the positive control, which defines the time scale over which the average of all intensity values is calculated. The average of fluorescence intensity values in the presence of each VHH normalized to the average of fluorescence intensity values for the positive control (100%) was defined as the percentage of aggregation, as shown in A.

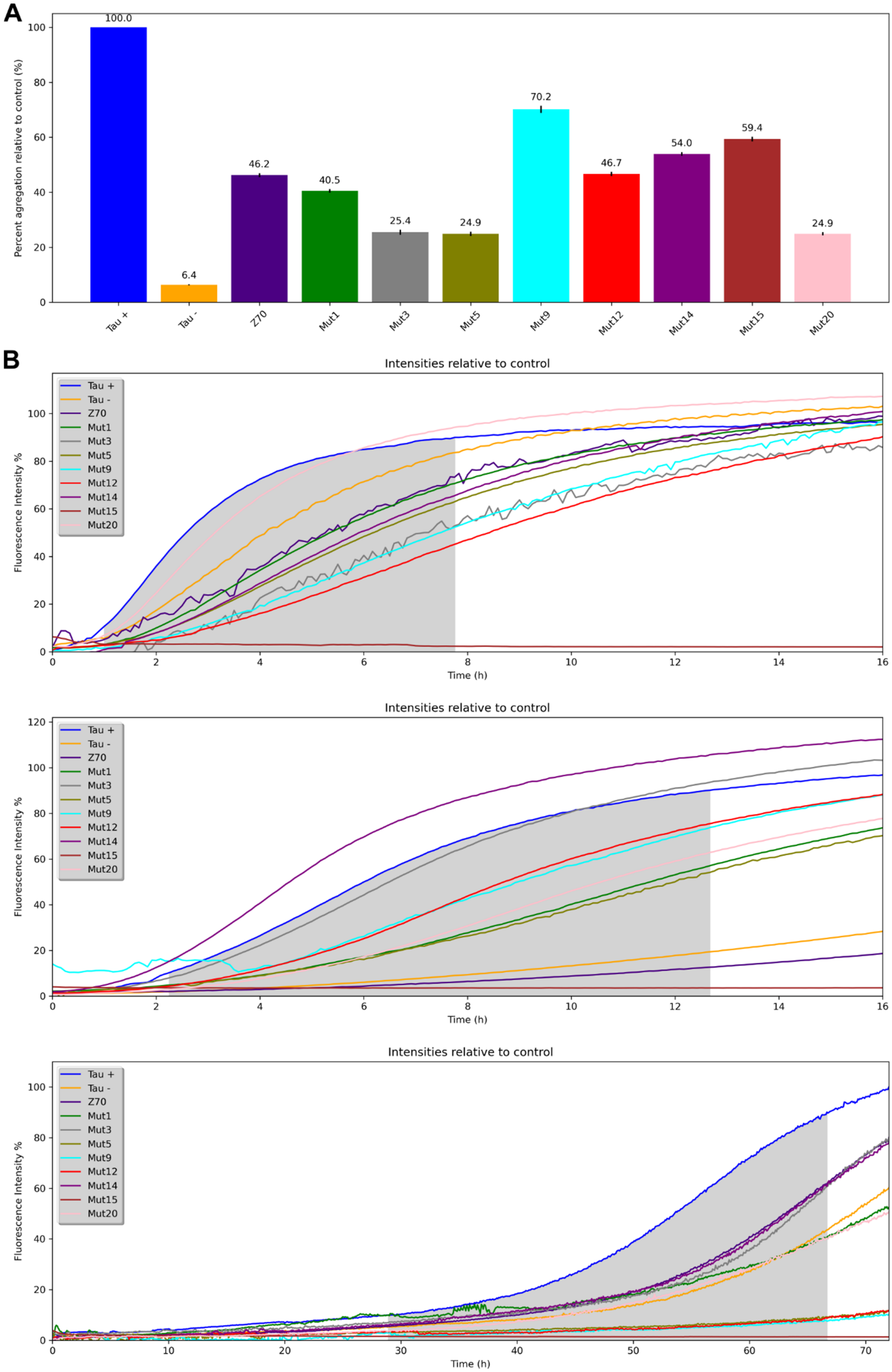
A
Percent agregation relative to control (%)
100.0
6.4
46.2
40.5
25.4
24.9
70.2
46.7
54.0
59.4
24.9
Tau +
Tau -
Z70
Mut1
Mut3
Mut5
Mut9
Mut12
Mut14
Mut15
Mut20
B
Intensities relative to control
Fluorescence Intensity %
Time (h)
Tau +
Tau -
Z70
Mut1
Mut3
Mut5
Mut9
Mut12
Mut14
Mut15
Mut20
Intensities relative to control
Fluorescence Intensity %
Time (h)
Intensities relative to control
Fluorescence Intensity %
Time (h)

Figure S11 Inhibition efficiency of different Z70-derived VHHs over Tau aggregation tested at a 0.2:1 VHH:Tau molar ratio. Positive and negative controls correspond to 10 µM Tau in the presence of 2.5 mM heparin (Tau +) and the absence of heparin (Tau -), respectively. The other conditions correspond to 10 µM Tau in the presence of 2.5 mM heparin and the different VHHs at 2 µM. **A.** The percentage of aggregation corresponds to the fluorescence intensity ratio between Tau in the presence of a VHH and Tau alone (Tau +, 100% aggregation) during the aggregation process. B. Intensity curves from *in vitro* aggregation assays from 3 independent experiments. Data are represented as mean ± sem, the mean value is written above the bars. The gray area corresponds to the 10-90% dynamic range of fluorescence intensity measured for the positive control, which defines the time scale over which the average of all intensity values is calculated. The average of fluorescence intensity values in the presence of each VHH normalized to the average of fluorescence intensity values for the positive control (100%) was defined as the percentage of aggregation, as shown in A.
